# Emotions in “the world”: cultural practices, products, and meanings of anger and shame in two individualist cultures

**DOI:** 10.3389/fpsyg.2013.00867

**Published:** 2013-12-05

**Authors:** Michael Boiger, Simon De Deyne, Batja Mesquita

**Affiliations:** ^1^Center for Social and Cultural Psychology, Faculty of Psychology and Educational Sciences, University of LeuvenLeuven, Belgium; ^2^Laboratory of Experimental Psychology, Faculty of Psychology and Educational Sciences, University of LeuvenLeuven, Belgium

**Keywords:** affordances, cultural practices, cultural products, semantic associations, anger, shame

## Abstract

Three studies tested the idea that people’s cultural worlds are structured in ways that promote and highlight emotions and emotional responses that are beneficial in achieving central goals in their culture. Based on the idea that U.S. Americans strive for competitive individualism, while (Dutch-speaking) Belgians favor a more egalitarian variant of individualism, we predicted that anger and shame, as well as their associated responses, would be beneficial to different extents in these two cultural contexts. A questionnaire study found that *cultural practices* promote beneficial emotions (anger in the United States, shame in Belgium) and avoid harmful emotions (shame in the United States): emotional interactions were perceived to occur more or less frequently to the extent that they elicited culturally beneficial or harmful emotions. Similarly, a *cultural product* analysis showed that popular children’s books from the United States and Belgium tend to portray culturally beneficial emotions more than culturally harmful emotions. Finally, a word-association study of the shared *cultural meanings* surrounding anger and shame provided commensurate evidence at the level of the associated response. In each language network, anger and shame were imbued with meanings that reflected the cultural significance of the emotion: while culturally consistent emotions carried relatively stronger connotations of emotional yielding (e.g., giving in to anger and aggressing against the offender in the United States), culturally inconsistent emotions carried relatively stronger connotations of emotional containment (e.g., a stronger emphasis on suppressing or transforming shame in the United States).

## INTRODUCTION

There are cultural differences in emotions that can be understood from the central goals in each culture. For example, comparative research in North America and Japan has shown that the most prevalent and intense emotions are those that fit the main cultural orientations in these contexts (Kitayama et al., 2006; Boiger et al., 2013). Anger, an emotion that marks individual entitlement and sets clear boundaries, was experienced more frequently in individualist North American contexts. In contrast, shame was experienced relatively more often in collectivist Japanese contexts, consistent with the fact that shame underlines a concern with social connectedness and fitting in. The prevalent emotions thus appear to be those that help to realize the respective cultural goals.

While most previous cross-cultural studies on emotion focused on differences in people’s psychological tendencies (e.g., emotion experience, expression, or perception; Matsumoto, 1990; Mesquita and Karasawa, 2002; Masuda et al., 2008), the current study will shift attention to the sociocultural level (i.e., the culturally shared practices, products, and meanings of emotion). By doing so, we answer the call by cultural psychologists to document cultural variation not only in terms of people’s psyches but also in terms of their specific cultural worlds (Morling and Lamoreaux, 2008; see also the mission statement of Frontiers in Cultural Psychology; Kurtis and Adams, 2013). Adopting the cultural psychology perspective that “the psychological ⋯ is grounded in and also fosters the sociocultural” (Markus and Hamedani, 2007, p. 3), we propose that the observed cultural differences in people’s emotions are afforded and scaffolded by differences in “the world” – for example, the cultural practices people engage in, the common artifacts they encounter, or the shared (linguistic) meaning systems they reference. We argue that people’s cultural worlds are structured in ways that promote and highlight those emotions and emotional responses that are beneficial in achieving the central goals of their culture.

In the present studies, we will show that the common cultural practices, products, and meanings in North American and Belgian contexts are geared toward promoting and highlighting culturally fitting or beneficial emotions; cultural practices, products, and meanings are crucial elements in the sociocultural constitution of the psyche (see Markus and Hamedani, 2007). We believe that research on how people’s cultural worlds facilitate and highlight *emotions* is particularly wanting: while studies on the structure of people’s everyday worlds have been burgeoning in other areas (e.g., on the cultural products of individualism–collectivism; for a review, see Morling and Lamoreaux, 2008), they are practically non-existent for emotion (for exceptions, see Tsai et al., 2007a; Boiger et al., 2013). In three studies, we will demonstrate respectively that the common interpersonal interactions, the popular children’s books, and the semantic associations within the respective languages, all foster and represent culturally valued levels and types of anger and shame. Moreover, by comparing two individualist cultures – the United States and Belgium – we want to bring attention to finer yet relevant distinctions within the cultural goal of individualism.

### MORE THAN ONE WAY OF DOING INDIVIDUALISM

Western Europe and North America have both been described as “Western” individualist cultures in contrast to countries such as Japan or China, which are considered “Eastern” collectivist cultures (e.g., Markus and Kitayama, 1991; Triandis, 1995). Although overarching tendencies toward the cultural goal of individualism in the former and toward collectivism in the latter appear to hold true, finer distinctions within these large-scale categories can be made (Oyserman et al., 2002; Kitayama et al., 2009). People’s engagement in individualism – broadly conceived as a system of ideas, values, and practices that emphasizes independent entities striving for autonomy (e.g., Hofstede, 1980; Markus and Kitayama, 1991) – appears to manifest itself differently in the U.S. and the Western European cultural context: while Americans endorse a *competitive* form of individualism, Western Europeans favor a more *egalitarian* variant of individualism (see Schwartz and Ros, 1995)^1^.

#### Competitive individualism in the united states

We refer to the U.S. variant of individualism as *competitive* because cultural ideals in U.S. (middle-class) contexts emphasize the value of standing out among others, having high self-esteem, and achieving personal success (Heine et al., 1999; see also Stephens et al., 2009). Although the last frontier officially disappeared over a century ago, the mentality of the self-sufficient frontiersmen has lived on in American cultural ideals such as the “American Dream” and the “pursuit of happiness” (Kitayama et al., 2009). For example, according to the American Dream ideology, everybody has equal opportunities at success and happiness; poverty is attributed to a lack of effort on the part of the poor, and thus justified (Hochschild, 1995).

Consistently, achievement and other self-enhancement values turn up as some of the highest ranking values in research with students and teachers from different regions of the United States (Schwartz and Ros, 1995; Schwartz and Bardi, 2001). The U.S. focus on mastery and hierarchy (e.g., ambition, success, wealth, social power) is achieved at the expense of harmony (e.g., equality, social justice, helping others; Schwartz and Ros, 1995). This is reflected in the high residential mobility in the United States, which underlines a striving for personal achievement at the cost of persistent relationships (Oishi, 2010). Finally, Americans also tend to place more value on the individual pursuit of happiness as compared to a promotion of independent ideas (Schwartz and Ros, 1995). These observations fit the proposition that Americans endorse a vertical form of individualism, which sees individuals as unequal in status and under social pressure to compete (Singelis et al., 1995; Triandis, 1995).

#### Egalitarian individualism in Belgium

We refer to the Belgian variant of individualism as *egalitarian* because cultural ideals in Belgium emphasize the integrity of the individual within a social network of equal rights (and not just equal opportunities; see Schwartz and Ros, 1995). The Western European history (and the Belgian history in particular) is one of overcoming differences and sacrificing individual interests for a greater cause. Compromising and finding solutions that work for several interest groups has been central for achieving the coherence of the Belgian state in the past, and continues to be so today (the so-called “Belgian compromise,” see, e.g., van de Craen, 2002). The Western European emphasis on egalitarianism has not only been immortalized in the motto of the French Revolution (“liberté, égalité, fraternité”) but is also reflected by the numerous welfare policies that support those who are less fortunate.

Consistently, Belgian (Dutch-speaking) students (*N* = 413; Boiger, unpublished data) rank universalism – an “understanding, appreciation, tolerance, and protection for the welfare of all people and for nature” (Schwartz and Bardi, 2001, p. 270) – as the second most important value (only preceded by benevolence, a universal first in value hierarchies around the globe; Schwartz and Bardi, 2001). Representative data from the European Social Survey show the same picture: universalism, security (i.e., “safety, harmony and stability of society, of relationships, and of self”; Schwartz and Bardi, 2001, p. 270), and tradition are ranked as 2nd, 4th and 5th in Belgium (ESS, 2008) – substantially higher than in the United States, where they rank at the bottom of the value hierarchy as 7th, 6th, and 9th (Schwartz and Bardi, 2001). The Belgian emphasis on persistent social relations is also reflected in a very low residential mobility in comparison to the United States (e.g., Long, 1991). These observations are largely consistent with the idea that Western Europeans endorse a horizontal form of individualism, in which individuals are seen as ultimately equal in status and under a stronger social pressure to conform (Singelis et al., 1995; Triandis, 1995).

### ANGER AND SHAME IN THE UNITED STATES AND BELGIUM

Cross-cultural studies on emotions in the United States and Western Europe are rare (but see Kitayama et al., 2009), especially compared to the body of research that established cultural variation between North America and East Asia (Mesquita and Karasawa, 2002; Kitayama et al., 2006; Tsai et al., 2007b). We propose that anger and shame may be well-suited candidates for a comparison between the United States and Belgium: while anger highlights individual entitlement – and may thus be an emotion that is rather consistent with the ideals of competitive individualism, shame highlights concern for others – which may play a relatively larger role in achieving the goals of egalitarian individualism.

The cultural significance of emotions such as anger and shame can be described at several levels (Mesquita, 2003). At the level of *emotional patterning*, there is quite a bit of evidence for the prevalence or intensity of those emotions that are conducive to cultural goals (and the relative absence of emotions that violate the central cultural goals; Kitayama et al., 2006; Boiger et al., 2013). At the level of the *emotional response* activation, there is support for the idea that the responses commonly associated with an emotion are commensurate with the cultural significance of the emotions. For instance, while anger comes with a tendency to *contain* the emotion in cultures that see anger as threat, it is associated with *yielding* to and expressing the emotion (e.g., stamping and yelling) in cultures that value anger (Mesquita and Frijda, 1992). In sum, it appears that emotions, whether at the level of the emotional patterning or at the level of the associated responses, are highlighted and promoted to the extent that they match the central cultural goals.

#### The cultural significance of anger

In general, anger signals the belief that others are blocking one’s goals, that one deserves more than one is getting, and that there is a chance of having one’s will if action is taken (Averill, 1982; Ellsworth and Scherer, 2003; Kuppens et al., 2007). In the U.S. cultural context of competitive individualism, anger is consistent with the cultural goal of competing against others and achieving high status: by experiencing and expressing anger, people assert their desires and pursue their goals in the face of obstacles. At the level of the emotional patterning, cultural practices in the United States appear to be geared toward up-regulating or promoting anger (Boiger et al., 2013). Anger is consequently an emotion that is encountered rather frequently in “the world” of Americans (Kitayama et al., 2006). At the level of the associated responses, an aggressive impulse may be relatively more common than, for example, a tendency to leave things as they are and withdraw (see Averill, 1982): by *yielding* to the emotion and engaging in antagonistic action – for example, in terms of verbal or physical aggression – anger fulfills the cultural goal of asserting one’s desires and pursuing one’s goals.

In comparison, in the Belgian context of egalitarian individualism, anger may play a more ambivalent role. On the one hand, by emphasizing a first-person perspective and an autonomous take on the world, anger is in line with the overarching cultural goal of individualism. On the other hand, by emphasizing one’s own desires (possibly over those of others), anger collides with the egalitarian aspect of Belgian individualism. At the level of the emotional patterning, anger may be an emotion that is neither particularly promoted nor avoided in Belgium: cultural practices may limit certain occurrences of anger to ensure smooth transactions between individuals, without avoiding anger altogether. Anger may be encountered, albeit less frequently than in the United States. At the level of the associated responses, an ambivalent urge to both assert oneself and withdraw from the situation may be common in Belgium; distancing may function as a regulatory response to *contain* the emotion and limit the potentially harmful consequences of anger (see Mesquita, 2003).

#### The cultural significance of shame

Shame brings attention to those areas in which one has failed in the eyes of others (Tangney, 1991; Tracy and Robins, 2004; Boiger et al., 2013; see also Bender et al., 2012) and thus highlights concern for the opinion of others. In the U.S. context of competitive individualism, shame is a highly undesirable emotion: by emphasizing personal flaws, shame undermines the cultural goal of standing out and achieving high self-esteem. At the level of the emotional patterning, cultural practices in the United States appear to steer clear of shame by down-regulating or avoiding shame; consequently, shame is encountered relatively rarely in “the world” of U.S. Americans (Cohen, 2003; Kitayama et al., 2006; Boiger et al., 2013). At the level of the associated responses, this stance toward shame may be reflected by a tendency to *contain* the emotion, e.g., by suppressing or transforming shame into a more acceptable experience (Allyn, 2004; Bear et al., 2009).

In comparison, shame is more consistent with the egalitarian emphasis on conformity and the maintenance of egalitarian relationships that are pursued in Belgium: by signaling when transactions with others have gone awry, shame may provide important information about what needs to be mended (Ha, 1995; Sznycer et al., 2012). At the level of the emotional patterning, shame may then be an emotion that is up-regulated or promoted, and that is encountered relatively frequently in Belgium. Moreover, if shame plays a role in restoring or repairing relationships in the Belgian context, there may be less of an urge to contain the emotion. At the level of the associated responses, the typical response may instead be to *yield* to the emotions and to capitalize on its relationships-restoring potential by seeking closeness with others (see Mesquita and Karasawa, 2004)^2^.

### OVERVIEW OF HYPOTHESES AND STUDIES

The goal of the present studies was to show that cultural practices, products, and meanings in “the world” reflect the cultural significance of anger and shame. Our overarching hypothesis was that cultural practices, products, and meanings in the United States and Belgium promote and highlight those emotional patterns and emotional responses that are beneficial for the cultural goals of competitive and egalitarian individualism (while avoiding those emotional patterns and emotional responses that violate these cultural goals).

We tested this hypothesis both for the emotional patterning and the associated responses. The emotional patterning was investigated in the first two studies: Study 1 examined the extent to which anger and shame are promoted and avoided in the social interactions that are commonly encountered in the United States and Belgium (which reflect *cultural practices*); Study 2 investigated the prevalence of anger and shame in popular children’s books from the United States and Belgium (which constitute a *cultural product*). We predicted that anger would be promoted and highlighted in the United States, whereas it would be less promoted (albeit not avoided) and limited in Belgium; moreover; we expected that shame would be avoided and limited in the United States, whereas it would be promoted and highlighted in Belgium. We operationalized cultural practices in terms of social interactions and cultural products in terms of children’s books because for both of these, culturally functional differences in emotional patterning have already been shown in North America and East Asia (Tsai et al., 2006; Boiger et al., 2013).

In Study 3, we tested our overarching hypothesis in terms of the *associated emotional responses*: making use of large-scale word-association data, we tested the hypothesis that culturally beneficial emotions are imbued with meanings that indicate relatively more yielding to the emotion (an aggressive response to anger, a closeness-seeking response to shame), whereas culturally inconsistent emotions are associated with meanings that indicate relatively more emotional containment (a distancing response to anger, a suppressing response to shame). The large scale of these word-association datasets allowed drawing conclusions about the system of meanings that is culturally shared by people with a common cultural background and language.

## STUDY 1: CULTURAL PRACTICES – AFFORDANCES OF ANGER AND SHAME IN SOCIAL INTERACTIONS

Study 1 investigated how cultural practices, that is, the “particular ways of acting and interacting in the recurrent episodes of everyday life” (Markus and Hamedani, 2007, p. 10), afford anger and shame in the United States and Belgium. In a referent-shift questionnaire, we asked people to tell us, for a random sample of anger and shame situations from their own culture, how frequently and intensely these situations would be experienced in their cultural context. We expected that cultural practices in the United States promote anger, such that highly angering situations are perceived to occur more frequently; in comparison, we expected that anger was neither particularly promoted nor avoided in Belgium, such that there is no clear association between what is perceived to occur frequently and what is perceived to be angering. For shame, we expected that cultural practices in the United States avoid shame, such that highly shameful situations are perceived to occur less frequently; in comparison, we expected that shame was promoted in Belgium, such that highly shameful situations are perceived to occur more frequently. We had already confirmed these predictions for the U.S. participants in a previous study (Boiger et al., 2013, Study 1). In Study 1, we replicated this previous study in Belgium and compared the Belgian data against the U.S. data from Boiger et al. (2013). We will report findings from both samples, but only the Belgian findings are new evidence for our hypothesis.

### SAMPLING SITUATIONS OF ANGER AND SHAME

We modeled the situation sampling procedure after our previous study in the United States. In both cultures, we first obtained a range of highly salient, autobiographical events through semi-structured in-depth interviews. We then complemented our pool of situations with daily experiences of anger and shame from experience sampling. We refer to Boiger et al. (2013) for a detailed description of the situation sampling in the United States.

#### Interviews

Semi-structured interviews were conducted with 37 (Dutch-speaking) Belgian students (19 women). All participants were born and resided in Belgium. The Belgian students (*M* = 18.7, SD = 1.02) were matched in age with the U.S. participants (*M* = 18.9, SD = 0.64) from our previous study, *t*(56) = 0.81, *p* = 0.42. As in the U.S. study, we asked respondents to narrate situations that fit particular types of transactions with the environment: we asked about situations of *offense* to elicit anger narratives, and about situations of *humiliation* to elicit shame narratives. Offense and humiliation have been used successfully in the past to elicit narratives of anger and shame (Mesquita, 2001) and they correspond to the “core relational themes” of anger and shame (Lazarus, 1991). The study yielded descriptions of 37 salient anger and 37 salient shame situations.

#### Experience sampling

Thirty-nine participants (24 women) completed a daily diary, in which they reported on their anger and shame experience for seven consecutive days. Again, we matched the Belgian (*M* = 19.6, SD = 1.7) and the U.S. (*M* = 19.0, SD = 4.9) participants in age, *t*(90) = 0.78, *p* = 0.44. The daily diary study in Belgium was slightly different from the previous study in the United States: in the United States, we had conducted an experience sampling of general emotional experience (four times a day for 7 days) and had then selected experiences related to anger and shame. Because the rate of anger and shame occurrences was low in the United States, we opted for the more targeted daily diary approach in Belgium. The Belgian daily diary study yielded 186 anger and 153 shame situations.

#### Scripting situations

We reduced all situation descriptions to short vignettes that retained the following elements: (1) the ongoing activity of the protagonist, (2) the relationship between the actors involved, and (3) the specific event that lead to the emotion (see Boiger et al., 2013). We excluded anger and shame situations that could not be reduced according to this script format (e.g., because essential elements were missing or because the situations were too complicated). The final sample of Belgian situation vignettes consisted of 144 anger situations and 137 shame situations. To make the situation vignettes salient for our target student populations, we named the protagonists according to the most popular names of their birth cohort, that is, students between the age of 18 and 23 (FOD Economie, unpublished data). In naming the protagonists we respected the gender of the respondent who had originally reported the situation.

### METHOD

#### Participants

Participants were 112 (Dutch-speaking) Belgian students (82 females). We required that all participants had to be born in Belgium or to have moved there before the age of 13; we excluded two participants who did not fulfill these criteria from the analyses. The Belgian participants were of Caucasian (93.6%), Asian (2.7%), ethnically mixed (1.8%), African (0.9%), and Arab (0.9%) descent. The Belgian participants (*M* = 18.7, SD = 1.2) were younger than their U.S. counterparts (*M* = 21.6, SD = 2.9) of the previous study, *t*(105.9) = 8.87, *p* > 0.001.

#### Referent-shift questionnaire

Two versions of the questionnaire were created, an anger and a shame version. For each version of the questionnaire, 20 anger/shame situations were randomly sampled from the final sample of Belgian situation vignettes; the selection was stratified by gender such that approximately half of the situations had initially been reported by women and half by men^3^. Participants indicated for each of the situations separately (a) how *frequently* it occurred in their culture (“How likely do most students you know experience a situation like this?”) and (b) how *powerful* the situation was to elicit the associated emotion (“How likely is it that a situation like this – if it were to happen – would lead most students you know to being angry/ashamed?”) on a 7-point Likert scale ranging from 0 (not at all likely) to 6 (extremely likely). We used a referent-shift format, asking participants about most students they know, because this format is less susceptible to self-presentational biases (see Kitayama et al., 1997; Chiu et al., 2010), which we expected to play a role when reporting on negative emotions (see Bender et al., 2012).

Anger and shame were defined in an introductory paragraph to the questionnaire in ways that included less intense experiences of the same emotion: “In this study we are interested in how and when people experience anger (this includes being angry, mad or annoyed with someone)” or “In this study we are interested in how and when people experience shame (this includes feeling humbled, feeling inadequate, or feeling embarrassed).” Because it had been deemed necessary to repeat this definition of shame when asking about the emotion-eliciting power of the situation in the previous U.S. questionnaire, we also repeated the definition in the Belgian questionnaire.

#### Procedure

Half of the participants completed the anger version of the questionnaire, half the shame version. In both versions, participants indicated for each situation its frequency and emotion-eliciting power. The material was created in English and then translated by native Dutch-speakers into (Belgian) Dutch. One of the authors, who is a native Dutch-speaker and fluent in English, checked all translations for accuracy.

### RESULTS

#### Preliminary analyses: establishing cultural consensus

To ensure that the U.S. and Belgian participants indeed reported on *shared* cultural practices, we first conducted consensus analyses (Romney et al., 1986). Consensus analysis establishes if there is agreement among participants, in this case on their perception of the frequency and emotion-eliciting power of the situations. Consensus analysis makes use of a factor analysis (principal component analysis, PCA) on the participants as units of analysis given their responses. Consensus is assumed to be present if the ratio between the first and the second eigenvalue is 3 or larger (Weller, 2007). We conducted separate consensus analyses for the Belgian and U.S. participants, using their combined ratings of situation frequency and power. The findings showed that both the American and Belgian participants drew on shared perceptions of the frequency and power of the anger situations (ratio_U.S._ = 3.0, ratio_Belgium_ = 6.5) and shame situations (ratio_U.S._ = 3.5, ratio_Belgium_ = 3.6) within their cultural group.

#### The situational promotion and avoidance of anger and shame

**FIGURE 1:**
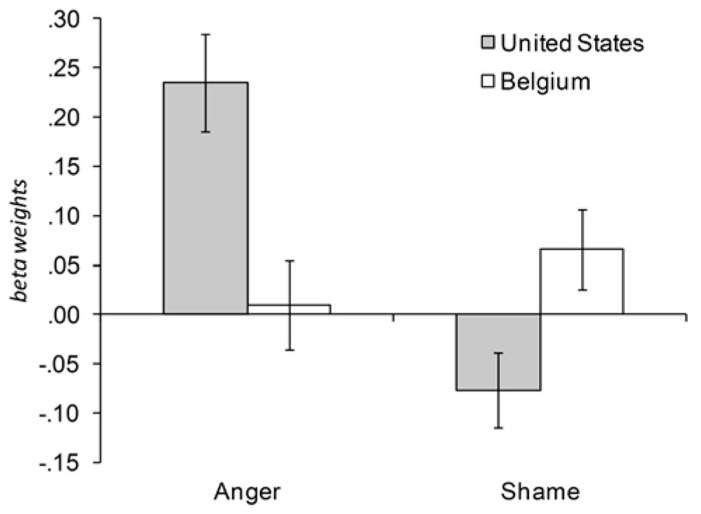
**Frequency of anger and shame situations as predicted by U.S. and Belgian students’ perception of the situations’ emotion-eliciting power**. Beta weights of random slopes predicting situation frequency from the situation’s emotion-eliciting power (multi-level models with situations nested in participants). U.S. data from Boiger et al. (2013). Error bars show standard errors.

We had predicted that anger was promoted in the United States, while it was neither promoted nor avoided in Belgium; we expected that shame was avoided in the United States, while it was promoted in Belgium. To test our hypotheses, we calculated multilevel regression models (with situations nested within participants) using the program MLwiN 2.27 (Rasbash et al., 2013). We tested our predictions about emotion promotion (that is, situations occur frequently to the extent that they elicit beneficial emotions) and avoidance (that is, situations occur rarely to the extent that they elicit harmful emotions) by regressing the perceived frequency of anger and shame situations on the emotion-eliciting power of these situations, allowing for random intercepts and slopes. We first calculated separate multilevel regressions for each culture. In a next step, we used the full dataset and entered culture as a level-2 predictor to test for cultural differences. **Figure 1** shows the promotion and avoidance of anger and shame in the United States and Belgium.

***Anger***. Consistent with our hypothesis, anger was promoted in the United States and neither promoted nor avoided in Belgium. As previously reported (Boiger et al., 2013), U.S. participants perceived U.S. anger situations to occur *more frequently* to the extent that they elicited stronger emotions (*b* = 0.24, *Z* = 4.7, *p* < 0.001)^4^. The Belgian data also supported our hypothesis. For the Belgian participants, who rated Belgian anger situations, the frequency of the situations was not related to the intensity of the elicited emotion: Belgian participants perceived anger situations to occur *neither more nor less frequently* to the extent that they elicited stronger emotions (*b* = 0.01, *Z* = 0.20, *p* = 0.84). This cultural difference between the United States and Belgium was significant as indicated by the situation power (respondent-mean centered) × situation culture (effect coded: -1 = United States, 1 = Belgium) interaction, *b* = -0.11, *Z* = 3.20, *p* < 0.01.

***Shame***. Our predictions were equally confirmed for shame: in line with our predictions, shame was avoided in the United States and promoted in Belgium. As previously reported (Boiger et al., 2013), the U.S. participants perceived U.S. shame situations to occur *less frequently* to the extent that they elicited stronger feelings of shame (*b* = -0.08, *Z* = 2.03, *p* < 0.05). The Belgian data also supported our hypothesis: Belgian participants perceived Belgian shame situations to occur *more frequently* to the extent that they elicited stronger feelings of shame (*b* = 0.07, *Z* = 1.65, *p* < 0.05, one-sided); again, this cultural difference was significant as indicated by the situation power (respondent-mean centered) × situation culture (effect coded: -1 = United States, 1 = Belgium) interaction, *b* = 0.07, *Z* = 2.50, *p* < 0.05.

### DISCUSSION

Study 1 has yielded evidence for differences in the cultural practices of anger and shame. Daily interactions (at least as perceived) promoted emotions that were consistent with the respective cultural goals (anger in the United States, shame in Belgium) and avoided emotions that violated cultural goals (shame in the United States). The fact that we found neither promotion nor avoidance of anger in Belgium suggests that anger is indeed ambivalent in this cultural context: some highly angering situations may occur frequently (e.g., situations with close others that do not pose an immediate threat to egalitarian relations) and others may be rare (e.g., situations that occur in public or school contexts where anger may be seen as harmful to egalitarian relations); which situations participants perceived as frequent and which situations they perceived as angering is then, across all situations, not significantly related. Future research may want to investigate if such situation-specific patterns of promotion and avoidance have led to the zero-association in Belgium. Indeed, similar research in other cultures has yielded evidence for the situation-specific promotion of anger and shame (Boiger et al., submitted).

One shortcoming of Study 1 is that it is not possible to fully disentangle whether the observed differences originated in the kinds of stories that were generated by U.S. and Belgian participants, or whether these differences are driven by differences in the ways U.S. and Belgian participants perceived the same events. *Post-hoc* analyses on the Belgian participants’ rating of the U.S. situations (which we had excluded for the purpose of the present study; see text footnote 3) revealed that the reported finding of shame promotion in Belgium only holds for Belgian situations and not for U.S. situations. The reported findings can thus not fully be explained by a Belgian perception “bias” alone. Although we do not have comparable data of U.S. participants rating Belgian situations, previous research with U.S., Japanese, and Turkish samples consistently yielded more pronounced patterns of anger and shame promotion (or avoidance) for own-culture than other-culture situations (Boiger et al., submitted; Boiger et al., 2013). Once again, these findings underline that the characteristics of the situations that people encounter in their culture play a role in the culture’s patterns of emotional experience. A further limitation of Study 1 is the use of self-report data of participants’ perception of what is frequent and powerful to elicit emotions in their culture. Future research may want to explore the occurrence of anger and shame situations in daily life with, e.g., experience sampling methods. In Study 2, we set out to provide convergent evidence for the cultural promotion of beneficial emotions by examining the portrayals of anger and shame in tangible aspects of people’s cultural worlds: the books that parents read to their children.

## STUDY 2: CULTURAL PRODUCTS – REPRESENTATIONS OF ANGER AND SHAME IN POPULAR CHILDREN’S BOOKS

Using a sample of popular children’s books from the United States and Belgium, we tested the hypothesis that commonly encountered cultural products highlight culturally consistent emotions and limit inconsistent emotions. We expected that U.S. children’s books are more likely to contain portrayals of anger than Belgian children’s books, while Belgian children’s books are more likely to portray shame than U.S. children’s books. We studied popular children’s books (including ones not initially created in the culture where they became popular), because those books are most widely distributed, and thus most likely to be encountered by children.

### METHOD

#### Selection of children’s books

In November 2008, we selected the 19 best-selling children’s books from each the United States and (Dutch-speaking) Belgium. In the United States, the books were selected based on sales lists published by amazon.com; in Belgium, the books were selected on the basis of sales lists issued by the Belgian bookseller Standaard Boekhandel as well as on the basis of recommendations of the Stichting Lezen [The Reading Foundation]. These were assumed to reflect the modal sources for parent’s choices in each country. We selected books for children up to 8 years, as this age-range covers some of the milestones of emotional development (e.g., Saarni et al., 1998). Further inclusion criteria were that (a) only the top-selling book for each author was selected and that (b) books had to contain a story with a plot or a collection of several short stories (that is, we did not include educational or craft books)^5^. The length of the selected texts (in terms of the number of words) did not differ significantly between the United States and Belgium^6^. A list of the children’s books included in Study 2 can be found in the Appendix.

#### Coding for anger and shame

Two trained judges coded all children’s books for instances of anger and shame, using ATLAS.ti 5.7 (Muhr, 2010). We defined anger and shame instances as those text segments that either contained explicit emotion words (anger: e.g., angry, mad, furious; shame: e.g., embarrassed, ashamed) or explicitly described emotional actions (anger: verbal/physical aggression, temper tantrums; shame: blushing, apologizing for a wrongdoing). The judges were instructed to focus on the actual wording of the text and to not make inferences. After an initial coding of 10 books – half from the United States, half from Belgium – disagreements in coding were discussed and additional inclusion/exclusion criteria added to the coding schedule (e.g., the inclusion of emotions during imagined or hypothetical episodes). For our analyses, we treated books as the units of analysis; that is, we determined how many books contained at least one reference to anger or shame. Inter-coder agreement for all books was 86.8% for anger (Cohen’s kappa = 0.70) and 97.4% for shame (Cohen’s kappa = 0.78).

### RESULTS

**FIGURE 2:**
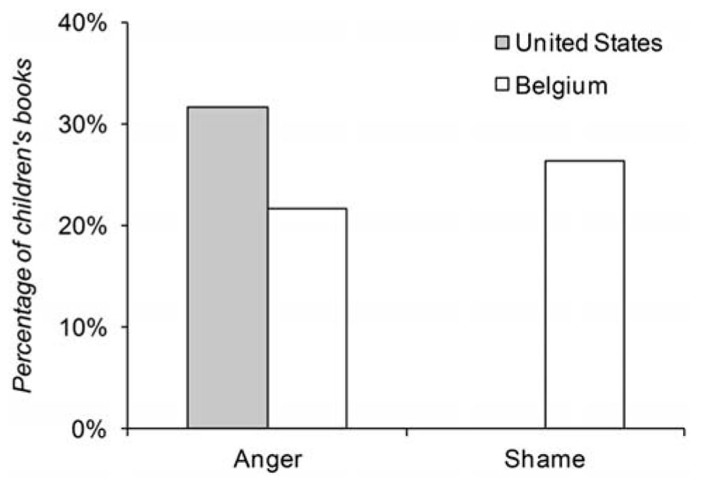
**Percentage of children’s books with anger or shame episodes**.

Building on our findings from Study 1, and in line with our hypothesis for the prevalence of culturally beneficial emotions, we predicted that children’s books from the Unites States compared to books from Belgium are more likely to contain instances of anger and that children’s books from Belgium compared to books from the United States were more likely to contain instances of shame. Because of the small sample size, we used Fisher’s exact tests (two-sided) to test these hypotheses. As shown in **Figure 2**, the percentage of children’s books that portrayed anger in the United States (31.6%) and Belgium (21.1%) differed in the expected direction; however, that difference was not statistically significant (*p* = 0.71). In line with our hypothesis, none of the American children’s books contained any reference to shame, while approximately one-fourth (26.3%) of the Belgian children’s books contained at least one instance of shame; this difference was statistically significant (*p* < 0.05).

We tested in a series of *post-hoc* analyses if American children’s books portrayed more anger than shame and if Belgian children’s books portrayed anger and shame to equal extents, using McNemar tests for paired samples (with continuity correction). We found that the American children’s books were more likely to contain portrayals of anger than shame, χ^2^(1) = 4.17, *p* < 0.05. In Belgium, the number of books that portrayed anger and the number of books that portrayed shame was not significantly different, χ^2^(1) = 0.00, *p* = 1.00. Moreover, in all the Belgian children’s books that contained an anger episode, we also identified a shame episode. In these books, shame tended to occur when protagonists realized the harmful consequences of their angry behavior.

### DISCUSSION

Study 2 partially supported the hypothesis that culturally beneficial emotions are highlighted in cultural products whereas harmful emotions are limited and contained. Our analysis of popular children’s books from the United States and Belgium showed both cross-cultural differences in the extent to which shame was portrayed as well as differences in the relative prevalence of anger and shame portrayals in the books from each culture. In line with our predictions, we found that U.S. children’s books compared to Belgian books were less likely to portray shame. Moreover, anger was more frequently portrayed than shame in the U.S. children’s books, while both emotions were equally frequent in the Belgian children’s books.

Against our predictions, we did not find evidence for the hypothesis that anger is more prevalent in U.S. cultural products than it is in Belgian cultural products. We may have not been able to detect differences between the United States and Belgium because of the limited number of books included in our samples and, consequently, the low power to detect effects. The fact that we did not find the predicted cultural differences may also be due to the particular cultural product that we investigated. It is possible that children’s books in the United States refrain from explicit portrayals of anger, while these anger portrayals may be rather common in media targeted at teenagers (see Wilson et al., 1990). Another explanation may be that books portraying anger are more popular in Belgium than expected because of the way anger is presented in these books: in the Belgian books in our sample, anger episodes always co-occurred with shame episodes. In these books, shame may act as a “socializing emotion” (Röttger-Rössler et al., 2013) that counteracts anger portrayals by highlighting its detrimental effects.

The pattern of findings in Study 2 hints to the idea that there may be specific meanings associated with anger and shame in the United States and Belgium. As noted above, it appears that anger in the Belgian children’s books had a negative connotation in that it was often set off with shame later in the story. Study 3 examined the idea that anger and shame are imbued with different meanings in terms of the associated responses. We suggested that these connotations at the level of the emotional responses are commensurate with the cultural goal of highlighting or limiting specific emotions.

## STUDY 3: CULTURAL MEANINGS – SEMANTIC ASSOCIATIONS OF ANGER AND SHAME

To test the hypothesis that the consistency of emotions with cultural goals is reflected in the connotations associated with the emotion, we used large-scale word-association datasets from the United States and Belgium. These large-scale word-association data provide approximations to the networks of meaning as available in the language of a particular culture (Nelson et al., 2004); in other words, they reveal the meanings people associate with using certain words. In Study 3, we examined the semantic associations of words in the categories of anger and shame. To this end, we selected a sample of central anger and shame words from the available cue words in these word-association datasets. We then established how closely related (or similar) these anger and shame words were to words that indicated emotional yielding on the one hand, and emotional containment on the other. We expected that culturally consistent emotions are relatively more strongly associated with responses that indicate yielding (an aggressive response to anger, a closeness-seeking response to shame), whereas culturally inconsistent emotions are associated with meanings that indicate emotional containment (a distancing response to anger, a suppressing response to shame).

Building on our ideas about the U.S. cultural goal of competitive individualism and the Belgian cultural goal of egalitarian individualism, we predicted that anger in the United States carries a meaning of wanting to aggress against the offender rather than distancing oneself from the situation; we expected that anger in Belgium is associated with connotations of both aggression and distancing. We expected that shame in the United States is associated with a meaning of suppressing or transforming the emotion rather than seeking closeness with others; we expected that shame in Belgium carries stronger connotations of closeness-seeking than emotion-suppression. In Study 3, we first focused on within-culture comparisons of emotional containment vs. yielding because the U.S. and Belgian word-association datasets differed in size and were thus not directly comparable; we then compared this *relative* emphasis on containment over yielding (or vice versa) across cultures.

### SAMPLING EMOTIONAL RESPONSE WORDS

To test our hypothesis about the semantic associations of anger and shame in the United States and Belgium, we needed samples of words that captured the emotional responses of aggression and distancing (for anger) and of suppression and closeness-seeking (for shame) in both languages. To this end, we re-analyzed the American and Belgian interviews (see Study 1). In these interviews, participants did not only report on the antecedent situations, but also on what they felt like doing and what they actually did when the situation happened. We first selected all segments in which participants talked about their emotional responses and then coded them for their content. Finally, we identified for each relevant segment the verb that best captured the respective response.

#### Selecting relevant segments

Two coders in each culture selected from the narratives of their own culture segments in which the participants talked about their emotional responses (that is, their impulses or action tendencies as well as their actual behavior), using the program ATLAS.ti 5.7 (Muhr, 2010). Action tendencies were defined as a wish, inclination, urge, or preference for a certain way of acting. Actual behavior was defined as the interviewee’s reported behavior in the situation. In both cultures, the judges agreed on more than 90% of the text selections for both action tendencies and action (agreement was established for a subset of the interviews). Any differences in the selection of text segments were discussed until agreement was reached.

#### Coding relevant segments

In a next step, two trained judges coded all relevant text segments for the emotional responses of interest. In the anger-section of the interview, we defined aggression as “fighting, physical aggression, or verbal aggression,” and distancing as “increasing the distance from the situation, another person, or a relationship.” In the shame-section, we defined suppressing the emotion as “not expressing or covering up the emotion” and seeking closeness as “wanting to be closer to the person, talking, or explaining.” Moreover, we coded for “doing nothing” in both sections in order to account for non-action. The coders were instructed to use only one of the codes for each segment and to focus on what the respondents actually said rather than making inferences.

Inter-coder agreement was above 90% (*M* = 94.9, SD = 4.2) across codes and cultures, with an average kappa of 0.82 (SD = 0.14) in the U.S. and 0.76 (SD = 0.20) in Belgium. When talking about anger, 95% of all participants referred at least once to aggression, distancing, or doing nothing, and when talking about shame, 76% of all participants referred at least once to suppressing the emotion, seeking closeness, or doing nothing; there were no cultural differences in the extent to which our coding system covered participants’ narratives. There were, however, cultural differences in the number of participants that reported these responses at least once. During anger episodes, Americans (90.5%) were more likely to report aggression than Belgians (48.6%), χ^2^(1) = 10.15, *p* < 0.001; in comparison, Belgians (48.6%) were tendentially more likely to report a tendency to distance themselves from the situation compared to Americans (23.8%), as indicated by a weak trend, χ^2^(1) = 3.45, *p* = 0.06. During shame episodes, Americans (33.3%) were more likely to report that they suppressed the emotion than were Belgians (8.1%), χ^2^(1) = 5.97, *p* < 0. 05; in comparison, Belgians (45.9%) were tendentially more likely to report a tendency to seek closeness with others than were Americans (23.8%), as indicated by a weak trend, χ^2^(1) = 2.78, *p* = 0.095.

#### Selecting words that reflect the predicted emotional responses

To select words that captured aggression and distancing (for anger) and suppressing and closeness-seeking (for shame), we referenced those text segments that had been coded with the respective responses. We then extracted the verb that, by itself, captured the interviewee’s response. We extracted 21 verbs related to aggression, 11 verbs related to distancing, 7 verbs related to suppressing or transforming shame, and 21 verbs related to seeking closeness. For all extracted verbs, we referred to dictionary translations into the respective other language and selected the nine (seven in the case of suppressing) words that (1) were mentioned most frequently in the interviews across both cultures and (2) were present as cues in both word-association datasets. We additionally included the preposition capturing the (relational) direction of the response (see Frijda et al., 1989): “against” for aggression, “away” for distancing, “not” for suppression, and “toward” for seeking closeness; we made sure that all of these had been mentioned by the interviewees in the respective text segments at least once. This brought the final selection of words to 10 for each emotional response in each culture (eight in the case of suppression). **Table 1** lists the final selection of emotional response words in English (with the Dutch equivalents in brackets).

**Table 1:** Selected cue words that reflected the predicted emotional responses in Belgium and the Unites States.

Anger		Shame	
Aggression	Distancing	Suppression	Closeness-seeking
yell [roepen]	ignore [negeren]	accept [aanvaarden]	talk [praten]
shout [gillen]	leave [weggaan]	cry [wenen]	answer [antwoorden]
argue [twisten]	run [weglopen]	defend [verdedigen]	stay [blijven]
hit [slaan]	go [gaan]	control [controleren]	explain [uitleggen]
slap [meppen]	avoid [vermijden]	laugh [lachen]	discuss [discussiëren]
hurt [bezeren]	disappear [verdwijnen]	leave [achterlaten]	chat [babbelen]
beat [kloppen]	escape [ontsnappen]	stop [stoppen]	listen [luisteren]
grab [pakken]	forget [vergeten]	not [niets]	hug [omhelzen]
punch [slagen]	withdraw [terugtrekken]		ask [vragen]
against [tegen]	away [weg]		toward [naar]

*Dutch equivalents are given in brackets.*

### METHOD

#### Participants

Both datasets initially included participants from countries other than the United States and Belgium (e.g., from the United Kingdom or the Netherlands). After exclusion of these participants, the final sample of participants used in the current study consisted of 38,497 U.S. Americans (21,912 females, 16,585 males) and 63,729 (Dutch-speaking) Belgians (42,162 females, 20,616 males, 951 unspecified). All participants had volunteered to participate in this study.

#### Material

In the U.S., participants provided associations to 7,006 cue words (59% nouns, 17% adjectives, 17% verbs, and 7% others); in Belgium, participants provided associations to 12,571 Dutch cue words (65% nouns, 17% adjectives, 16% verbs, and 2% others). The cue words in both samples were iteratively sampled, starting with an initial selection of cue words that were then continuously enlarged by adding the most frequently given associations as new cue words.

#### Procedure

The procedure for the continued association task is described in detail in De Deyne et al. (2013). At early stages of the study, a small percentage of participants was recruited among first-year students at Dutch-speaking Belgian universities in exchange for course credit. The majority of American and Belgian participants were then recruited online and were invited to share the link to the study with their friends and family. All participants were asked to indicate their native language (e.g., British or American English, Dutch as it is spoken in the Netherlands or Belgium/Flanders), which allowed us to select participants from the United States and Belgium.

The participants were presented with lists of typically 14 cue words and were instructed to provide the first three words that spontaneously came to their mind. These list of cue words were selected randomly from the sample of cue words, such that each participant was presented with a different list of words. Participants were instructed that the three associations all had to be related to the initial cue word rather than forming a sequence of associations. For both the Dutch and American word-association dataset, a cue × cue network was derived that encoded only associations that were part of the cue set; this network was used for all subsequent analyses^7^.

#### Selecting anger and shame words

In order to capture the categories of anger and shame in each culture, we sampled anger and shame words from the available cue words. We identified candidates by making use of published lists of emotion word stems for anger and shame (Storm and Storm, 1987); additionally, we used a snow-ball technique to investigate the associative networks surrounding the identified anger and shame words. We included only words that were present in both datasets (according to dictionary translations). **Table 2** shows the nine anger and eight shame words that we identified in both datasets.

**Table 2:** Selection of anger and shame words in Belgium and the United States.

Anger				Shame			
United States		Belgium		United States		Belgium	
Cue word	Centrality ln (PageRank)	Cue word	Centrality ln (PageRank)	Cue word	Centrality ln (PageRank)	Cue word	Centrality ln (PageRank)
**anger**	27.98	kwaadheid	3.80	**ashamed**	1.38	**beschaamd**	4.21
**angry**	33.71	**kwaad**	55.69	**bashful**	1.74	**verlegen**	15.37
**annoy**	6.51	ergeren	3.85	disgrace	1.32	schande	2.06
frustration	4.35	frustratie	10.21	embarrass	1.36	generen	2.37
fury	4.53	**woede**	24.19	**guilt**	4.76	**schuld**	11.54
**hate**	29.81	**haat**	12.13	**humble**	2.98	**nederig**	2.72
irritating	5.13	**irritant**	25.61	humiliate	1.11	vernederen	1.86
**mad**	24.47	**boos**	54.48	**shame**	5.53	**schaamte**	8.27
offense	1.64	belediging	2.07				

* 
The five most central words within the network (according to PageRank) are shown in bold.
*

In a next step, we reduced the selection of anger and shame words to the five most relevant words for each emotion in each culture. We measured relevance in the network with the Page-Rank algorithm (Page et al., 1998; see also Griffiths et al., 2007); relevance according to PageRank is higher for words that have been frequently associated with words that also have many associations. Five words seemed a good compromise between being overly inclusive or restrictive, as the PageRank statistic tended to decrease after four to five words.

**Table 2** displays the relevance for all anger and shame words; the five most relevant anger and the five most relevant shame words in each culture are printed in bold. Because the relevant anger or shame words could differ between cultures, the final selection did not entirely overlap between the United States and Belgium. However, the two anger words that differed between the two cultures were highly similar, as “woede” is an accepted dictionary translation of “anger,” and “irritant” and “annoy” refer to the same underlying concept of annoyance. We used this reduced selection of relevant anger and shame words for all further analyses.

#### Analytic strategy

We examined the associations between emotions and responses by calculating pair-wise similarities between each emotion word and each associated emotional response word for each language separately. These similarities were calculated as cosines between the response frequency distributions and varied between 0 and 1; the response frequencies were log-transformed and were limited to the cue × cue adjacency matrices^8^. To test our hypotheses about the different meanings associated with anger and shame in each culture, we conducted permutation tests using Monte-Carlo approximations based on 100,000 random permutations. For example, to test our hypothesis that American anger is more strongly associated with a meaning of yielding (that is, aggression) than containment (that is, distancing), we calculated the observed average difference between the anger-aggression similarities and the anger-distancing similarities. We then compared the observed average differences against the distribution of the permuted values, which allowed us to establish a confidence interval around the simulated differences and to assign a *p*-value to the observed values. This permutation strategy was then also applied to establish the confidence intervals of the effect for the relative differences between U.S. and Belgian participants (that is, as a permutation-test over a difference of differences).

### RESULTS

#### The meaning of anger in the United States and Belgium

**FIGURE 3:**
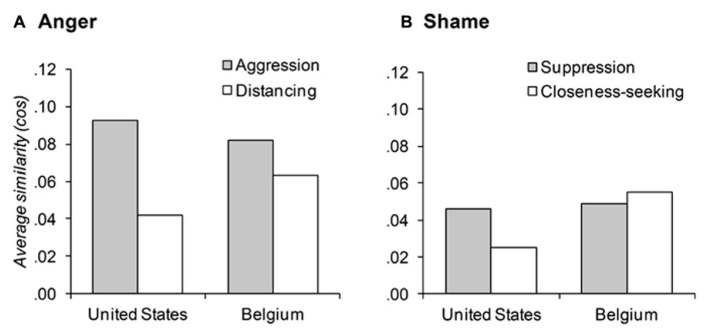
**Similarity between emotion words and words reflecting emotional responses in the United States and Belgium**.

**Figure 3A** shows the average similarities between the most relevant anger words and the words reflecting aggressive or distancing responses in the United States and Belgium. We found that anger had a strong connotation of aggression in the United States, while it included a relatively stronger connotation of wanting to distance oneself from the situation in Belgium. In line with our predictions, the selected anger words in the United States were significantly more similar to words that reflected aggression compared to words that reflected distancing from the situation, *d*(aggression, distancing)_U.S._ = 0.051, 95% CI [-0.013, 0.013], *p* < 0.001. Inconsistent with our hypothesis, anger in Belgium was also significantly more similar to words that reflected an aggressive response as compared to a distancing response, *d*(aggression, distancing)_Belgium_ = 0.018, 95% CI [-0.013, 0.013], *p* = 0.02. However, the emphasis on aggression over distancing was significantly larger in the United States than Belgium, *d*(*d*_U.S._, *d*_Belgium)_ = 0.032, 95% CI [-0.015, 0.015], *p* < 0.001.

#### The meaning of shame in the United States and Belgium

**Figure 3B** shows the average similarities between the selected shame words and the words that reflect suppressing or closeness-seeking responses. We found that shame had a strong connotation of wanting to suppress the emotion in the United States, while it included an equally salient connotation of wanting to engage in the relationship and seek closeness in Belgium. In line with our predictions, the selected shame words in the United States were significantly more similar to words that reflected a tendency to suppress the emotion compared to words that reflected a tendency to seek closeness, *d*(suppression, closeness)_U.S._ = 0.021, 95% CI [-0.009, 0.009], *p* < 0. 001. Inconsistent with our predictions, the selected shame words in Belgium were as similar to words that reflected suppression as they were to words that reflected closeness-seeking, *d*(suppression, closeness)_Belgium_ = -0.005, 95% CI [-0.010, 0.010], *p* = 0.41. However, the emphasis on suppression over closeness-seeking in the United States was significantly larger than in Belgium, *d*(*d*_U.S._, *d*_Belgium)_ = 0.026, 95% CI [-0.011, 0.011], *p* < 0.001.

### DISCUSSION

Study 3 made use of a hitherto untapped resource for studying cultural differences in the implicit meanings associated with specific emotions. By analyzing the semantic associations of anger and shame in large-scale word-association datasets from the United States and Belgium, we identified the meanings implicitly associated with those emotions in the respective languages. We expected that emotions that are consistent with U.S. and Belgian cultural goals carry meanings that reflect emotional yielding (an aggressive response to anger, a closeness-seeking response to shame), whereas culturally inconsistent emotions are associated with meanings that indicate emotional containment (a distancing response to anger, a suppressing response to shame).

Our findings confirmed our predictions for the United States. Anger in the United States carried a relatively stronger meaning of emotional yielding (that is, a stronger association with aggression than distancing), while shame carried a relatively stronger meaning of emotional containment (that is, a stronger association with suppression than closeness-seeking). In Belgium, our findings pointed to a more nuanced pattern of meanings than we had initially expected. We found that, contrary to our predictions, anger in Belgium also carried a primary meaning of emotional yielding (that is, a stronger association with aggression than distancing); however, this emphasis on emotional yielding over containment was smaller than in the United States. It is not entirely surprising that Belgian anger words would share aspects of the U.S. meaning – they were, after all, accepted dictionary translations of each other and aggression may have accounted for the lexical equivalence (see Mesquita, 1993). What seems to distinguish the meaning of anger between the United States and Belgium is that Belgian anger contains a relatively stronger secondary connotation of wanting to contain the emotion. This secondary connotation of Belgian anger also reflects the interviewees self-reported accounts in the preliminary study.

We equally did not find support for the prediction that shame would be primarily associated with emotional yielding in Belgium. Instead, we found that shame had a somewhat ambivalent meaning in Belgium: both emotional containment and yielding (that is, suppressing the emotion and seeking closeness) were equally strongly associated with shame. Although the primary connotation of shame did not differ between the United States and Belgium, the secondary connotation mattered more in Belgium than in the United States. In other words, shame carried a meaning of not wanting to feel the emotion in both cultures, but it had a stronger connotation of acknowledging its relation-restoring potential in Belgium.

## GENERAL DISCUSSION

Three studies provided evidence that the cultural practices, products, and meanings in “the world” afford and reflect emotional patterns and emotional responses that are consistent with the respective cultural goals. Based on the idea that U.S. Americans strive for a competitive form of individualism, we had predicted that anger is consistent with U.S. cultural goals, while shame violates these cultural goals. To the extent that Belgians favor an egalitarian form of individualism, we had predicted that anger is neither unequivocally beneficial nor harmful to Belgian cultural goals, while shame benefits these cultural goals.

Study 1, a referent-shift questionnaire about the commonly experienced anger and shame situations in the United States and Belgium, demonstrated that cultural practices appear to promote culturally beneficial emotions (anger in the United States, shame in Belgium) and to avoid culturally harmful emotions (shame in the United States); emotions that were neither culturally beneficial nor harmful were found to be neither promoted nor avoided (anger in Belgium). Study 2, an analysis of the prevalence of anger and shame in popular children’s books from the United States and Belgium, provided convergent evidence: cultural products in the United States tended to highlight anger, while cultural products in Belgium tended to highlight relatively more shame. Again, the culturally ambivalent role of anger in Belgium became evident in the finding that, in those books that contained an anger episode, anger portrayals were accompanied by portrayals of (the ensuing) shame in Belgium.

Study 3, a large-scale study of the semantic associations of anger and shame, found that the meanings associated with anger and shame are commensurate with the patterns we found at the level of the emotions: while the American meaning of anger reflected emotional yielding (i.e., aggression), Belgian anger carried a relatively stronger connotation of containing the emotion (i.e., distancing). In comparison, the U.S. meaning of shame was primarily about emotional containment (i.e., suppressing shame), while the Belgian meaning of shame contained a relatively stronger connotation of yielding to the emotion and acknowledging its potential for restoring relationships (i.e., seeking closeness with others). It thus appears that the cultural meanings at the level of the emotional responses complement the cultural affordances at the level of emotional patterns; cultural particularities at each level contribute to the overall cultural goals in a “redundant” fashion (Levy, 1978).

When taking these findings together, a cohesive picture of how “the world” affords and represents emotions emerges: cultural practices promote emotions that are in line with the cultural goals while avoiding or “down-regulating” emotions that violate these goals; our findings suggest that this occurs, for example, in terms of the social interactions that people encounter in their culture. Cultural products reflect these tendencies by portraying beneficial emotions more frequently than harmful emotions; these products reflect the intentional choices of both their creators and consumers (the latter being more likely in our case, where the sample of children’s books was selected based on popularity) and may contribute to the socialization of the cultural practices observed. Finally, the meaning systems embedded in people’s respective language reflect the consistency of emotions with cultural goals: culturally beneficial emotions carry relatively stronger meanings of emotional yielding, while harmful emotions carry relatively stronger meanings of emotional containment.

The present study set out by adopting the cultural psychology perspective that “the psychological ⋯ is grounded in and also fosters the sociocultural” (Markus and Hamedani, 2007, p. 3). In three studies we have shown that, indeed, the sociocultural highlights emotions and emotional responses that are congruent with the respective cultural goals. We hope that this study inspires future emotion research on the mutual constitution of the psychological and the sociocultural. This may, for example, entail studies on the actual *processes* of mutual influence (see also Morling and Lamoreaux, 2008). Taking a cultural and contextualized approach to emotions may also safeguard research against losing sight of what emotions really are: powerful connections between inner psyches and outer worlds.

## Conflict of Interest Statement

The authors declare that the research was conducted in the absence of any commercial or financial relationships that could be construed as a potential conflict of interest.

## Footnotes

1 We do not mean to suggest that a cultural group engages in only one cultural goal. A number of cultural goals beyond competitive and egalitarian individualism may be pursued simultaneously in the United States and Belgium. Potential candidates are the cultural goals of positivity, modernity, power distance, or uncertainty avoidance (see, e.g., Lamoreaux and Morling, 2011).

2 The relationship-restoring function of shame has mainly been discussed in the context of collectivist cultures (e.g., Mesquita and Karasawa, 2004; but see also Dijk etal., 2009). While we assume that shame plays a role for restoring or repairing relationships in both collectivist and egalitarian individualist contexts, the specific relational failures highlighted by shame may vary: in line with different ideas about selfhood, shame in collectivist cultures may be more frequently experienced in response to failures at being a “good” group member (Ha, 1995), while, in a context of egalitarian individualism, it may more commonly be felt in response to individual strivings that crossed another person’s interests.

3 The Belgian participants in the present study additionally rated the same (American and Japanese) situations as the participants in the previous study by Boiger etal. (2013). For the purpose of the present study, we excluded these other-culture situations.

4 The U.S. parameter estimates and standard errors reported here vary slightly from those reported in the original study by Boiger etal. (2013). This is due to the fact that we only included own-culture situations in the present study, whereas Boiger etal. (2013) compared own- and other-culture situations; the level-2 covariance matrices differed accordingly, leading to slightly different estimates. These differences are minimal and do not affect the pattern of significant results.

5 Due to an error during the selection process, two books by the same author (see Appendix, Capucilli, 2000, 2005) were included in the U.S. sample. Removing the second, less popular, book by Capucilli, (2000, “*The Potty Book for Girls*”) does not change the pattern of results, and we therefore decided to retain the book in all analyses.

6 Because of its length, we decided to code only parts of the book “*Pluk van de Petteflet*” (see Appendix, [Bibr B97]). We included the first three chapters, three chapters from the middle of the book, and the three last chapters; those were deemed sufficient to capture the essence of the story. The included text, which contained 11,000 words, was nonetheless substantially longer than the Belgian average for the remaining books (*M* = 640.44, *SD* = 823.1).

7 In the full datasets, each cue word was presented to at least 100 participants. However, because we excluded participants who were not from the United States or Belgium, the number of associations differed slightly for each cue in our study. To mitigate the different number of associations per cue, the total summed associative strength for each cue was normalized to sum up to one.

8 We obtained a similar pattern of results when using the cue × response adjacency matrices or *t*-score transformed response frequencies (see Church etal., 1991).
